# Directional control of neurite outgrowth: emerging technologies for Parkinson's disease using magnetic nanoparticles and magnetic field gradients

**DOI:** 10.1098/rsif.2022.0576

**Published:** 2022-11-09

**Authors:** K. Dhillon, K. Aizel, T. J. Broomhall, E. Secret, T. Goodman, M. Rotherham, N. Telling, J. M. Siaugue, C. Ménager, J. Fresnais, M. Coppey, A. J. El Haj, M. A. Gates

**Affiliations:** ^1^ Healthcare Technologies Institute, Department of Chemical Engineering, University of Birmingham, Birmingham, UK; ^2^ Institut Curie, PSL Research University, CNRS, Sorbonne Université, Physico Chimie, Paris, France; ^3^ Sorbonne Université, CNRS, Laboratoire Physicochimie des Électrolytes et Nanosystèmes Interfaciaux, PHENIX, 75005 Paris, France; ^4^ School of Pharmacy and Bioengineering, Guy Hilton Research Centre, Keele University, Staffordshire, UK; ^5^ School of Medicine, Keele University, Staffordshire, UK

**Keywords:** magnetic nanoparticles, regenerative medicine, tissue engineering, neurite outgrowth

## Abstract

A challenge in current stem cell therapies for Parkinson's disease (PD) is controlling neuronal outgrowth from the substantia nigra towards the targeted area where connectivity is required in the striatum. Here we present progress towards controlling directional neurite extensions through the application of iron-oxide magnetic nanoparticles (MNPs) labelled neuronal cells combined with a magnetic array generating large spatially variant field gradients (greater than 20 T m^−1^). We investigated the viability of this approach in both two-dimensional and organotypic brain slice models and validated the observed changes in neurite directionality using mathematical models. Results showed that MNP-labelled cells exhibited a shift in directional neurite outgrowth when cultured in a magnetic field gradient, which broadly agreed with mathematical modelling of the magnetic force gradients and predicted MNP force direction. We translated our approach to an *ex vivo* rat brain slice where we observed directional neurite outgrowth of transplanted MNP-labelled cells from the substantia nigra towards the striatum. The improved directionality highlights the viability of this approach as a remote-control methodology for the control and manipulation of cellular growth for regenerative medicine applications. This study presents a new tool to overcome challenges faced in the development of new therapies for PD.

## 1. Introduction

Parkinson's disease (PD) is a neurodegenerative condition that arises during the progressive degeneration of midbrain dopaminergic (mDA) neurons in the substantia nigra pars compacta (SNc). The loss of mDA neurons results in the loss of connections between the SNc and striatum. While pharmacological treatments exist for the short-term replacement of dopamine in the SNc-striatal circuit, there is a critical clinical need for cell therapies to restore dopaminergic cells in the SNc [1–4]. Potential cell therapies for this condition also depend upon the ability of the transplanted cells to reinnervate with neurons in the striatum. However, the rate of regeneration in the central nervous system is slow [5,6] which limits recovery. This technically challenging aspect of neuronal cell engineering is crucial for successful cell integration, circuit reconnection and ultimately disease remission. Several approaches to directing neuronal growth have been investigated, including the use of mechanical cues and magnetic nanoparticles (MNPs) to generate tension to guide outgrowth [7–9]. While the study of neuronal outgrowths has been investigated over a long period of time, the control of outgrowth direction is a relatively newer approach that offers ways of progressing potential cell therapies for neurodegenerative conditions.

Within the regeneration process growth cones are extended from the neuronal cell which are capable of generating forces through their motion, which can aid in growth and repair [10]. Combining the mobility of these growth cones and biological cues enables neuronal outgrowths to become directed to a target region [11]. Therefore, it is considered that other approaches to generating directional cues could help achieve more complete reinnervation. One potential approach to generating topological gradients and directional cues is through the use of MNPs guided by magnetic fields [6,8,9,12,13]. MNPs are a suitable candidate for applying mechanical forces to neuronal cells, being widely used in biomedicine settings, such uses as MRI contrasts [14–16], and are commercially available, with previously demonstrated biological safety.

MNPs have attracted attention as force carriers for the manipulation of cells owing to their ability to be controlled remotely through externally applied magnetic fields. Through the application of magnetic fields, the resulting forces can be applied either internally or externally to a cell, for example: through internalization into endosomes, or by binding to receptors and ion channels at the cell membrane. This approach has been recently used in a variety of cell and tissue engineering applications to regulate cell signalling pathways, control cell differentiation and promote tissue repair [12,17,18]. Commonly, varying magnetic fields are employed to create a field gradient (varying in time) to act upon MNPs [19]. However, static magnetic fields can also be designed to vary in space and therefore offer another method to generate sufficient field gradients to induce forces acting on MNPs.

**Figure 1.**
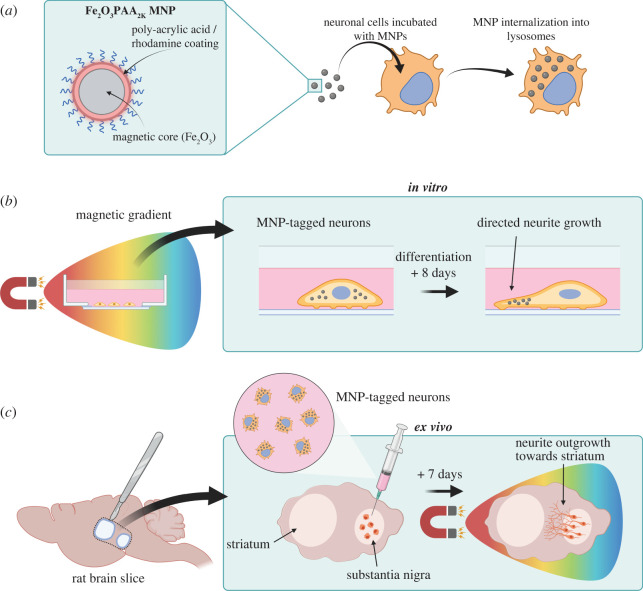
(*a*) Schematic of the experimental set-up. Poly-acrylic acid/rhodamine functionalized MNP with an Fe_2_O_3_ core was synthesized and incubated with PC12 cells to allow internalization. (*b*) The MNP-tagged cells were cultured in differentiation media while being exposed to static magnetic fields in order to direct neurite extension along the magnetic field gradients. (*c*) MNP-tagged neurons were also injected into rat brain slices modelling the nigral-striatal pathway. Magnetic fields were then used to direct neurite extension from the cells at the injection site towards the striatum.

To study the efficacy of using MNPs and static magnetic fields to help direct the neuronal outgrowths, we employ PC12 cells with MNPs and used a magnetic gradient to magnetically control neurite extension while undergoing differentiation (figure 1*a,b*). PC12 cells are a well-established model neuronal cell line in PD research. When cultured with neurotrophic factors, PC12 differentiate into neuron-like cells with neuronal morphology and express neuronal markers [20,21]. We then tested the translational potential of this approach in rat organotypic brain slices, which were used as a model of the SNc-striatal circuit. Using this system, we injected primary rat embryonic ventral midbrain (VM) dopaminergic precursor cells, which had been pre-labelled with MNP, into the SNc of rat brain slices. We then orientated the slices under the magnetic field gradient to promote directed cell and neurite outgrowth from the transplant region towards the striatum (figure 1*c*). After 8 days, we assessed cell and neurite outgrowth under magnetic control. The overall aim of this research was to demonstrate controlled directional neurite outgrowth in neuronal cells, which was validated using mathematical models. This technology provides proof of principle for magnetic control of neuronal outgrowth with clear applications for neurodegenerative cell therapies.

## 2. Methods

### 2.1. Computational methods

The magnetic field profiles were simulated by employing a finite-element (FE) approach using the commercial software *COMSOL v5.1.* Magnetic fields and the resulting gradients were simulated by employing the *Magnetic Fields, No Currents* toolbox, with initialization parameters (remnant magnetization *B*_R_) matching those of the neodymium iron-boron (NdFeB) magnets used in the experimental device (table 1). To calculate the directional motion of MNPs, the resulting field profiles were combined with the *Particle Tracing for Fluid Flow* toolbox with MNPs simulated as particles with diameter, mass and magnetic susceptibility (χ) matching those of experimentally employed MNPs (table 1).

**Table 1.** Parameters used for magnetic and MNP simulations.

simulation property	value	units
*B*_R_	1.42	T
MNP diameter	8.4	nm
MNP density	5240	kg m^−3^
*χ*	4.27	dimensionless

The magnetic susceptibility (*χ*) of the MNPs is of key importance when calculating the forces acting on a particle. The MNPs chosen for this study were iron-oxide particles coated with PAA (Fe_2_O_3_-PAA_2K_). The MNP diameter is 8.4 ± 1.9 nm with saturation magnetization values of 51–57 emu g^−1^ [12]. These particles have previously been reported to have good levels of internalization and stable magnetization within biological systems over long time periods [22]. From a Langevin function of magnetization, the magnetic behaviour of these particles is easily reproduced, and the susceptibility was calculated to be 4.27 from the change in magnetization at zero field.

In the instance of an MNP located within static magnetic field the force acting upon it while in a fluid medium can be found from the following equation [23]:

2.1 $$F_{M}=2\pi r_{p}^{3}\mu_{0}\mu_{p}\frac{\mu_{p}-\mu_{f}}{\mu_{p}+2\mu_{f}}\nabla H^{2},$$

where *r_p_* is the radius of the particle (in m), *μ_p_* and *μ_f_* is the permeability of the MNP and surrounding fluid, respectively, (where *μ* = 1 + *χ*) and ∇H is the magnetic field gradient in (A m^−2^). The motion for each MNP was calculated using the magnetophoretic force (equation (2.1)) applied across the sample region with the fluid medium taken to be water (*μ_f_* = 1) as an analogy to the MNP being located either in the cell or culture medium. MNP motion simulations included viscous drag and were simulated for a total time period of 8 h.

### 2.2. Magnetic device

**Figure 2.**
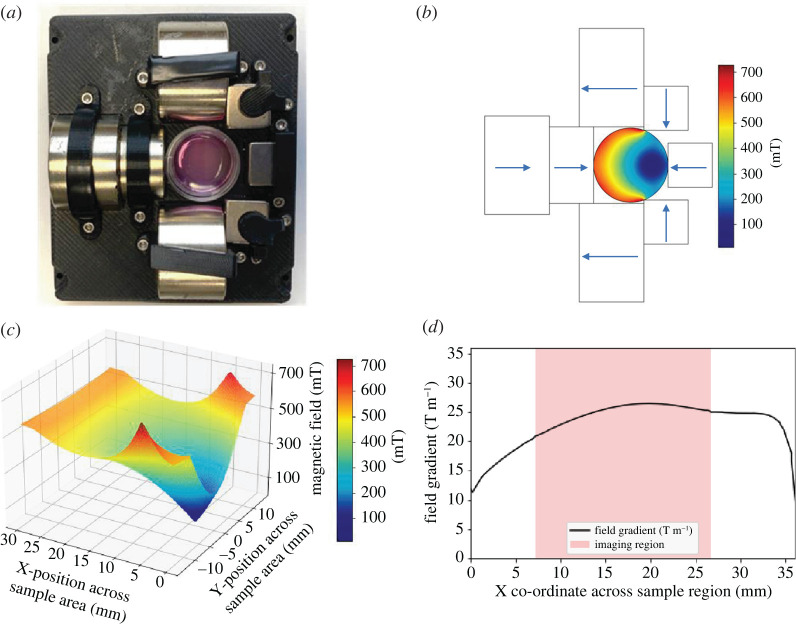
(*a*) Shows assembled device with sample. (*b*) Shows a schematic of the device highlighting the orientation of the permanent magnets and the simulated magnetic field. (*c*) Three-dimensional map of magnetic flux density across the sample area confirmed a steep gradient across the sample region. (*d*) Shows the resulting magnetic field gradient, and the imaging region highlighted demonstrates that in the sample region investigated the field gradient is greater than 20 T m^−1^.

To enable forces to be applied upon MNPs, a magnetic field (and gradient) needs to be established over the sample region. To achieve this, a device consisting of NdFeB permanent magnets (N42 grade) was created (as shown in figure 2*a*; electronic supplementary material, figure S1). The magnetic field was simulated using a FE method and the resulting magnetic field profile is shown in figure 2*c*, ranging from field strengths of approximately 700 to approximately 70 mT. The magnetic array device was mapped in three dimensions using an M3D-2A-PORT magnetic field mapper (SENIS) at a scan resolution of 200 µm (x,y) and a step size of 0.5 mm (z). The measured field map is shown in figure 2*c*; the values of magnetic field and the field gradient are found to align with the simulated magnetic field. The magnetic field gradient found along the magnetic field orientation is shown in figure 2*d* and is found to be greater than 20 T m^−1^ across the sample region.

### 2.3. Cell culture

The rat adrenal phaeochromocytoma cell line, PC12 (CRL-1721), obtained from ATCC, were cultured in suspension in basal medium made from Dulbecco's modified Eagle's medium (Gibco) supplemented with 10% horse serum (heat inactivated), 10% fetal bovine serum, 1% penicillin-streptomycin and 1% L-glutamine. Cells were kept in incubator at 37°C and 5% CO_2_.

### 2.4. Magnetic nanoparticles labelling

Cells were labelled with rhodamine-fluorescent Fe_2_O_3_-PAA_2K_ MNPs which were previously described and characterized by Bongaerts *et al.* [12]. The MNPs had a mean hydrodynamic size of 30.1 nm and were superparamagnetic in nature with a saturation magnetization of 55.7 emu g^−1^. Cells were labelled in suspension at a concentration of 2 mM (iron) in pre-warmed complete basal medium for 24 h prior to seeding.

### 2.5. PC12 differentiation

For differentiation into a neuronal phenotype, PC12 cells were seeded at 20 000 cells cm^−2^ density on 1% poly-L-lysine (Sigma) and 10 µg µl^−1^ laminin (Sigma L2020) coated glass-bottom dishes 24 h before initiating differentiation. Cells were differentiated for 8 days in culture with differentiation medium: Dulbecco's modified Eagle's medium (Gibco) supplemented with 5% horse serum (heat inactivated), 1% penicillin-streptomycin, 1% L-glutamine and 100 ng µl^−1^ nerve growth factor (recombinant human β-NGF 450-01, Peprotech) with media replenishment every 2 days.

### 2.6. Live/dead staining

PC12 cells were incubated with MNP as above and cultured in neuronal differentiation media under a static magnetic field with peak flux density of less than 200 mT over 8 days. Cell viability was then assessed after 8 days using a ReadyProbes™ cell viability assay (ThermoFisher) according to the manufacturer's instructions.

### 2.7. Rat brain slice preparation

Organotypic slice cultures were prepared from postnatal day 5–8 rat pups according to the interface method as previously described [18,24]. Briefly, pups were terminally anaesthetized by intraperitoneal injection of pentobarbital in accordance with UK Animals (Scientific Procedures) Act 1986. Sagittal brain sections of 300 µm were cut at 10° angles using a vibratome (Leica). Following removal of the cortex and hippocampus, slices were placed onto PTFE membranes with 0.4 µm pores to allow access to nutrients from below and maintain gaseous exchange from above.

### 2.8. Injection of magnetic nanoparticle labelled ventral midbrain cells

Slices were cultured for 7 days (with SN removed) before transplantation of E14 VM cells to the SN region of the slice. The day before transplantation, the VM was dissected from E14 rat embryos, dissociated and resuspended in medium containing 8 mM Fe_2_O_3_-PAA_2K_ MNPs (iron concentration). The suspension was added to the well of a plate and incubated overnight. The MNPs prevented the cells from adhering to the plate without affecting cell viability. The next day, 200–500 nl of MNP-loaded VM cell suspension was pipetted into the SN region of the slice. The slice was then transferred to a Petri dish and orientated within the magnet device so that the striatum was exposed to the largest flux density in order to promote cell/neurite growth towards the striatum. The magnetic device was housed within a cell culture incubator at 37°C, 5% CO_2_. Control slices were cultured in six-well plates at 37°C, 5% CO_2_ under no magnetic field. Slices were fixed 7 days following cell transplantation and immuno-stained for tyrosine hydroxylase (TH) to visualize both the cell bodies and process of dopamine neurons in the tissue and transplant.

### 2.9. Imaging

Light and fluorescent images were taken with EVOS M5000 or EVOS XL core microscopes (Thermofisher Scientific). Three-dimensional Z-stack imaging was conducted using Olympus Fluoview FV1000 confocal microscope. Neurite angle measurement was performed using the angle measurement tool in FIJI ImageJ2 software.

### 2.10. Analysis of rat brain slices

Images for each slice were stitched to form a montage, then rotated so that the striatal-SN axis was in the same plane for each sample. Each slice image was then divided into a grid of 20 regions of interest (ROI's) covering three regions as shown in electronic supplementary material, figure S2a. These regions consisted of the implant area, the striatal region and an outgrowth area located between the implant area and the striatum. For each sample, the mean grey value of each ROI in the outgrowth region was measured. These values were corrected by subtracting the mean grey value of the slice background due to staining from the background slice tissue. The orientation of TH+ cells and neurites present in the outgrowth region was also assessed using a directionality plug-in in ImageJ. This was used to calculate the relative average amount of TH+ structures at each orientation (±90°) within each sample. Only samples which contained TH+ cells and or neurites in the outgrowth region were included in the analysis.

### 2.11. Statistical methods

All statistical analysis was completed using Stata 17.0.

For two-dimensional neurite growth measurements, the measured angles were transformed to be a deflection relative to the field direction irrespective of lateral travel (i.e. ignoring left/right direction, with the key metric being the deflection angle). This results in measured deflection angles in the range of 0 − 90°. To evaluate the distributions in both control and MNP group, a Levene test for variance and a Shapiro test for normality were performed. It was found that the two distributions had equal variance and were both non-normally distributed (Levene test: *p* > 0.05, Shapiro test: *p* < 0.05). From the description of these distributions, a Kruskal–Wallis test was chosen as a suitable statistical test.

For pixel intensity analysis of slice TH staining, data are presented as medians +/- interquartile range (IQR). Statistical significance at 95% confidence level was applied using a Mann–Whitney test (two-tailed, unpaired), *p* < 0.05 was considered statistically significant.

## 3. Results

### 3.1. Simulation of magnetic nanoparticle motion

The direction of force exerted on MNPs within the sample region can be simulated to gain understanding as to how the external magnetic field and resulting gradient will drive directional neurite outgrowths. The force acting on an MNP and therefore to the attached sub-cellular structures and organelles in a static magnetic field can be calculated by employing a magnetophoretic model (equation (2.1)). The magnetophoretic model results in MNP motion along the direction of applied force.

Particle trajectories were simulated in a water medium over a time period of 8 h as an indication to the resulting directional outgrowth. Viscous drag forces were included to better understand the magnitude of forces exerted on the MNPs. The angle between the final position and the initialization position relative to the field direction was calculated for each simulated MNP.

**Figure 3.**
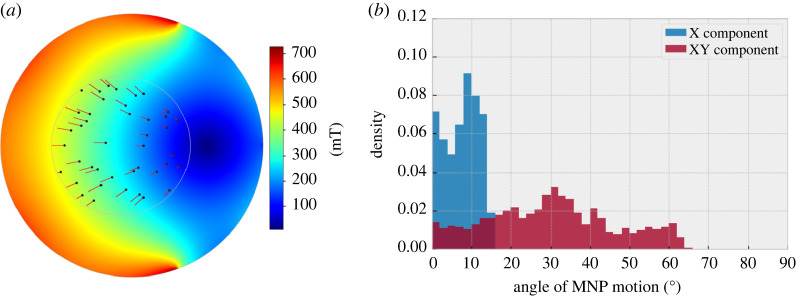
(*a*) Shows simulated trajectories of MNP motion within the magnetic device. Initial MNP positions (black dots) were randomized to the same sampling area used for two-dimensional imaging studies (grey circle). MNP motion was simulated over an 8 h period and the resulting angle of motion is shown in (*b*). Simulated MNP motion is included for both only including the X-component of magnetic field compared with including both X and Y components.

Initial particle positions were randomized to within a sub-region of the entire sample region which represented the locations available for imaging in experimental studies (indicated by the grey circle in figure 3*a*, black dots identify initial positions for a random sampling of particle positions). A total of 1500 particles were initialized with a resulting 1147 particle positions having a final position within the sampling region. The resulting angle of MNP motion is shown in figure 3*b*, it is of note that the majority of particles do not move along the horizontal (along the direction of the applied field) but have a small angular offset in the vertical direction (perpendicular to the main applied field direction). This can be seen from comparing simulations that only include forces created through the application of *B*_x_ components, to those in which *B*_x_ and *B*_y_ are included in the force calculation (figure 3*b*). When only *B*_x_ components of magnetic field are included, the applied force and resulting directional motion of MNPs show a narrower range of deflection from the direction of applied field. The resulting width of the peak indicates the field gradient is not uniform across the sample area in *B*_x_. Furthermore, when both the *B*_x_ and *B*_y_ components are included, the resulting MNP motion becomes more varied. In this scenario, the median angle is found to be 30.35°. From this, it is clear the combination of field gradients, not just the field gradient along the main direction of applied field are fundamental for the resulting directional force experienced by the MNP and their subsequent motion.

### 3.2. Neurite growth in a two-dimensional culture

**Figure 4.**
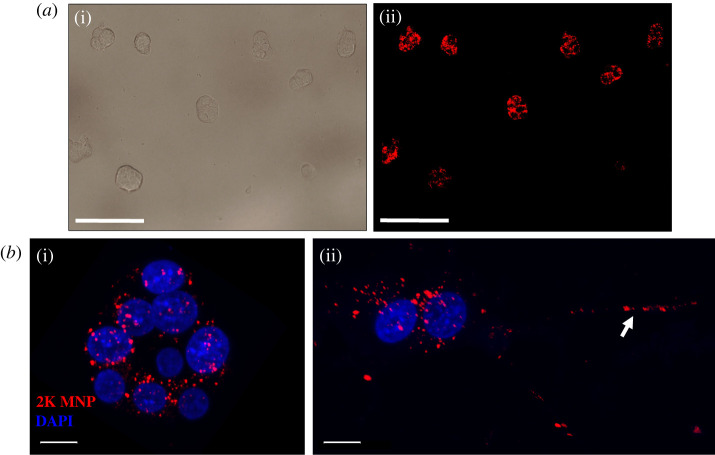
(*a*) PC12 cells labelled with rhodamine-fluorescent Fe_2_O_3_-PAA_2K_ MNPs (scale bar = 100 µm) (*b*) Z-stack (0.2 µm slices) confocal images of PC12 cells labelled with AA2K-MNPs at day 1 (i) and day 3 (ii) after differentiation, with white arrow showing MNPs in neurite extension (scale bar = 10 µm).

**Figure 5.**
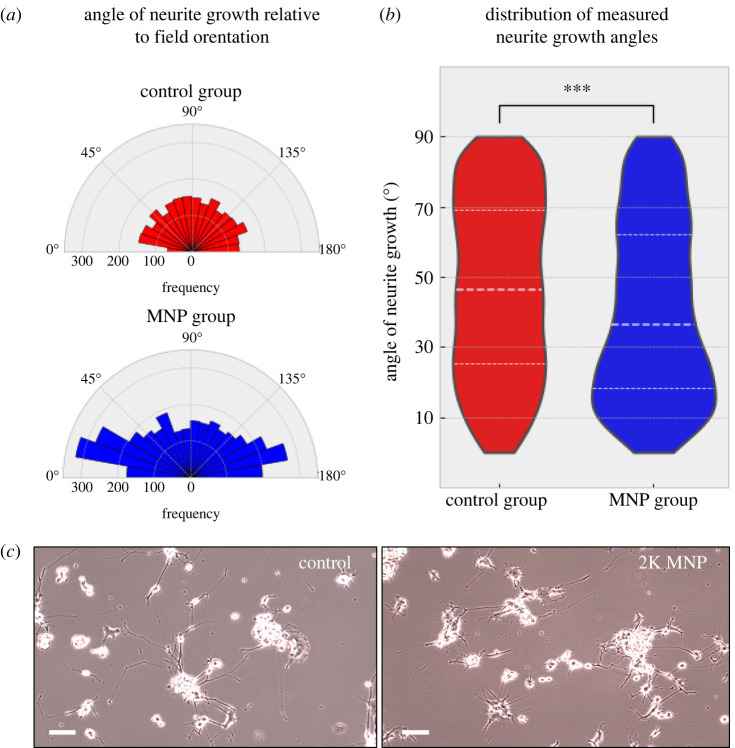
(*a*) Shows the range of measured neurite growth angles (*Θ*) from the applied field direction where the field direction is along the *x*-axis (0° and 180°). (*b*) Violin plot showing angle of deflection from the applied field direction between control and MNP groups (red and blue, respectively). With the dashed lines representing the median and IQRs of each group. (*c*) Brightfield images of control and 2K-MNP-labelled PC12 neurons after 8 days differentiation (scale bar = 200 µm).

PC12 cells labelled with Fe_2_O_3_-PAA_2K_ MNPs (figure 4*a*) were differentiated within the device (MNP group) and non-labelled cells within the device (control group) for a period of 8 days to encourage neurite growth. Fluorescent microscopy confirmed the presence of MNP after 1 day (figure 4*b*(i)), with MNP still detectable after 3 days of differentiation (figure 4*b*(ii)). Cell viability in response to MNP and magnetic field gradients was assessed using a live/dead assay. Results showed no effect of either MNP or magnetic field gradients on cell viability (electronic supplementary material, figure S2). We next assessed the effect of MNP and magnetic gradients on directional neurite outgrowth *in vitro*. From repeat incubations (MNP group *N* = 4, control group *N* = 3), images of cells with neurite outgrowths were taken from the imaging region (sample area simulated in figure 3*a*); summary statistics can be found in table 2. For each cell with neuronal outgrowths, the direction of growth was calculated as an angular displacement from the direction of applied magnetic field. The distribution of measured angle of neurite outgrowth is shown in figure 5*a*,*b*. The distribution of measured angles within the control group shows no preferential direction. However, the distribution found from the MNP group shows more favoured growth directions towards 0° and 180°. The resulting distribution of angles found from the MNP is skewed to the lower values, as can be seen from figure 5*b*, and the differences in median and IQR (dashed lines).

**Table 2.** Description of control and MNP groups for neurite growth analysis, and the resulting angle of growth (median and IQR).

	control group	MNP group
*N*	3	4
*n*	2487	3394
median (IQR)	46.332 (25.084, 69.075)	36.345 (18.138, 62.103)

From the descriptive data the median angle of deflection from the direction of field has been reduced from the control group value of 46.332 (IQR: 25.084, 69.075) to the device group value 36.345 (IQR: 18.138, 62.103). A Kruskal–Wallis test shows a significant difference between control group and MNP group with *p* < 0.001. Therefore, it has been shown that the influence of a static MNP on MNP-labelled cells can significantly increase the directional growth of neurites in two-dimensional slices.

### 3.3. *Ex vivo* rat brain slice model

**Figure 6.**
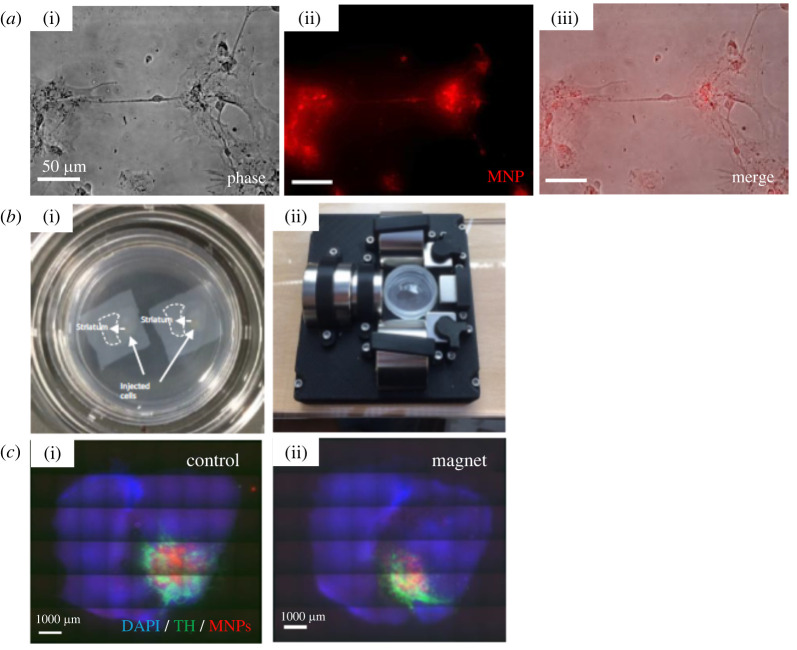
Live imaging of plated VM cells revealed good loading of the MNPs by VM cells. Phase contrast image is shown in (*a*(i)), fluorescent MNPs are shown in (*a*(ii)), and merge is shown in (*a*(iii)). Representative images of *n* = 4 are shown. Brain slices were cultured in Petri dishes (*b*(i)) before loading into the magnetic gradient device, with the striatum exposed to the largest magnetic flux density in the system (*b*(ii)). Fluorescent microscopy of injected slices confirmed MNP loading of VM cells and localization at the transplant site in both control (*c*(i)) and magnet groups (*c*(ii)). The slice is visible through DAPI staining (blue), VM cells are shown through TH staining (green) and MNP are shown through rhodamine fluorescence (red). Images representative of *n* = 4–6.

Following the *in vitro* experiments, we then tested the magnetic system in a more complex *ex vivo* rat brain slice model using primary VM cells labelled with Fe_2_O_3_-PAA_2K_ MNPs. We first confirmed MNP loading in VM cells using live cell imaging (figure 6*a*). Phase contrast and fluorescence microscopy (figure 6*a*(i–iii)) confirmed MNP loading in cells after 24 h of incubation. Rat brain slices were then loaded into Petri dishes (figure 6*b*(i)) before injection with the MNP-labelled cells. Slices were then placed within the gradient magnetic device for 7 days with the striatum positioned closest to the largest flux density to encourage cell growth from the implant site towards the striatum (figure 6*b*(ii)). Fluorescent microscopy of the slices confirmed TH+ cell bodies and MNP at the transplantation site after 7 days in both groups indicating the presence of transplanted dopaminergic neurons (figure 6*c*(i,ii)).

**Figure 7.**
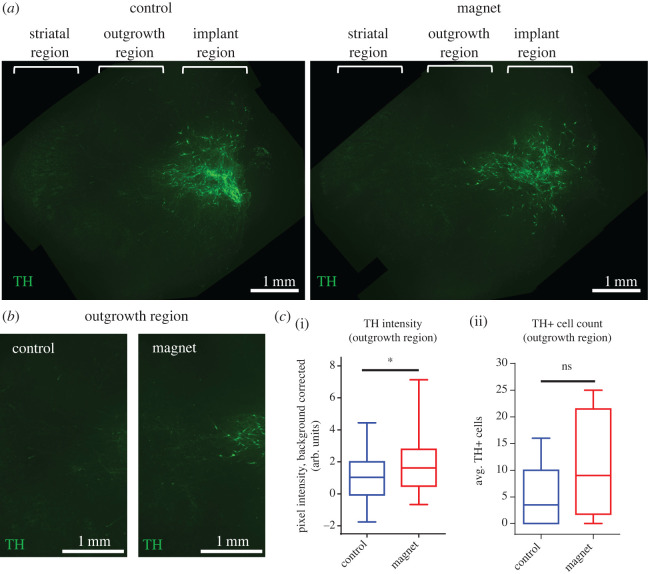
Magnetic gradient promotes neurite outgrowth in brain slices. Fluorescent images of TH-stained slices were divided into three regions (striatal, outgrowth and implant). Fluorescent microscopy of the outgrowth regions indicated that the magnetic device enhanced cell and neurite growth into the outgrowth region compared with the control region (*a*(i,ii)). Representative images are shown. Pixel intensity analysis in the outgrowth regions confirmed a significant increase in TH staining in the magnet group relative to the control (*b*). Values represent background corrected median pixel intensity; asterisk (*) represents *p* < 0.05. Error bars represent IQR with min. and max. values. *n* = 4 (magnet), *n* = 6 (control).

Next, we quantified the degree of TH +ve cell and neurite outgrowth away from the injection site in the presence or absence of the magnetic field gradient. This was achieved by analysing the amount of TH+ staining distal to the interface between the transplant and host explant boundary (figure 7*a*). The control groups showed relatively less outgrowth reaching the striatum, whereas slices exposed to the magnetic device showed a trend for denser outgrowth (figure 7*b*). For analysis, each slice was divided into zones comprising the injection zone, striatal zone and an outgrowth zone located between the injection and striatal zone (electronic supplementary material, figure S3a). The intensity of TH staining within the outgrowth zones was then quantified. Though there was no significant difference in the number of TH+ cells migrating into the proximal portion of the outgrowth zone of the host tissue (figure 7*c*(i,ii)), results showed a significant increase in TH staining intensity in this region in the magnet group. Though there is the possibility that even a non-significant rise in the number of TH+ neurons migrating into this region could increase the over TH+ staining, the significant increase in the staining in the magnet group was interpreted as being an indication that the overall neurite outgrowth in this group is relatively higher than in the control group (figure 7*c*; electronic supplementary material, figure S3b). We also analysed the directionality of the TH+ cells and neurites present in the outgrowth zone using the directionality ImageJ plug-in (electronic supplementary material, figure S3c(i)). Results showed a small shift in the orientation of the MNP-loaded cells and neurites when exposed to the magnetic field (trend only), with these cells and neurites aligning more towards the upper striatum. By contrast, the cells and neurites in the control group (where present) tended to align towards the lower striatum (electronic supplementary material, figure S3c(ii)).

## 4. Discussion

Directional control of neuronal processes is one of the long-standing challenges in neuronal tissue engineering. This technical barrier must be overcome before cell therapies can be harnessed for treatment of neurodegenerative diseases. Multiple recent studies, including from our group, have demonstrated the potential for MNP and magnetic fields to control cell signalling and influence direction of neurite outgrowth *in vitro* [6,9,18,25]. For instance, Semeano *et al.* recently demonstrated that MNP embedded in collagen-based coatings can stimulate microtubule expression, promote neurite elongation and can influence cell migration when combined with static magnetic fields [26]. Bongaerts *et al.* recently used Fe_2_O_3_-PAA_2K_-MNP and static magnetic fields provided by arrays of magnetized pillars to direct cell migration and induce neurite outgrowth in differentiated cells in the direction of the pillars [12]. In the current study, we created an asymmetric array of permanent magnets that provided a steep magnetic field gradient over a 3 cm region. We used this array to promote directional outgrowth of neurites using the same Fe_2_O_3_-PAA_2K_-MNP which were internalized in neuronal cells. In both studies, Fe_2_O_3_-PAA_2K_-MNP were non-specifically targeted and passively taken up by cells. Within this study, we also tested the short-term toxicity of the MNP and magnetic fields. Importantly, neither MNP nor static magnetic fields had any noticeable effect on cell viability, which is a critical consideration when developing MNP-based treatments. The application of static magnetic fields in both studies imparted non-specific tensile forces to cells to directly influence the directionality of neurite outgrowth. This effect is to be expected given the crucial role that mechanical forces play in control of cell polarity and neurite outgrowth [27]. Having said this, the precise downstream mechanisms through which the MNP-induced forces are transduced to cellular structures to alter neurite directionality require further study.

We also compared neurite outgrowth angles with MNP directional motion predicted using mathematical models. FE analysis simulations were performed to investigate the magnetic field gradients and resulting directional motion of MNPs. The simulation of the magnetic field gave field profiles and resulting field gradients that closely resembled that of the configured device. The resulting directions of motion are not directly parallel to the applied magnetic field (which would result in a 0° deflection) and are indicative that the combination of magnetic field vectors and molecular guidance signals from growth factors (such as nerve growth factor) secreted from neighbouring neurons play competing roles in the resulting neurite outgrowth direction [11,28].

Despite the small discrepancy between predicted MNP direction and observed neurite outgrowth direction, we still demonstrate that application of the magnetic gradient leads to a significant difference in the experimental outgrowth direction. The change in preferential outgrowth direction broadly mapped the changes in magnetic field gradient across the sample. It is of note that the MNP-labelled group had a median direction of 36.35°, compared with the median direction of motion from simulations of 21.53°. It is noted that the mathematical modelling approach demonstrated here does not include biological factors such as cell-binding and competing forces which may arise from biological mechanisms, including cues from neighbouring cells. However, these values measured are consistent with other mechanistic models found in literature which suggest the resulting growth direction would be approximately 30° due to the combination of magnetic vector directions [9].

Finally, we investigated the effect of our magnetic gradient device in a more complex *ex vivo* brain slice model using primary neurons to test the translational potential of our approach. Our experiments demonstrated that primary neuronal cells can be loaded with MNPs without inducing toxicity, and neuronal cell transplants can be tracked on brain slices for at least 7 days. These properties are important to consider in neuronal tissue engineering, which requires minimal MNP toxicity and maximal cell survival over time. Crucially, our results showed that magnetic gradients can promote dopaminergic outgrowth of MNP-labelled cells towards the striatum. Previous studies have shown it is possible to remotely trigger cell signalling and direct neuronal stem cells to sites of injury *in vivo* using MNP and magnetic guidance systems. These approaches were shown to facilitate neuronal differentiation and brain repair as well as alter neuronal electrophysiology and alter animal behaviour, respectively [29,30]. To our knowledge, the work presented here is the first study to demonstrate magnetically controlled cell and neurite outgrowth in primary dopaminergic cells transplanted into a complex *ex vivo* model. It should be noted that the magnetic field gradients used in our system (greater than 20 T m^−1^) are an order of magnitude greater than the field gradients currently used in the clinic. For example, MRI scanners use field gradients in the region of 5–50 mT m^−1^ [31], while larger field gradients (500 mT m^−1^) have been used for related *in vivo* studies [32]. The magnetic device within this study was designed to exhibit substantially steeper field gradients, as these would be sufficient field gradients to elicit a strong enough directional force on individual MNPs. These high field gradients are required to enable interaction of MNPs and magnetic field across the large physical distance between the substantia nigra and a permanent magnet located at the skull surface. While the current study confirms proof of principle for our technique, an alternative magnetic device, with similar magnetic field profiles, would be necessary before the approach could be translated to the clinic. Future work will focus on magnetic field optimization to promote further increases in directional neuronal outgrowth, but the ability to control cell behaviour in this way is nonetheless an important step which will ultimately be required for translation of neuronal cell therapies to the clinic.

Neurodegenerative conditions such as PD urgently require new therapies; however, directional control of neurite extension is one critical factor that is preventing clinical uptake of cell therapies for these diseases. In this work, we have demonstrated remote-controlled neurite outgrowth from neuronal cells *in vitro* and in an *ex vivo* model of the nigral-striatal pathway using MNP and magnetic gradients. Our experimental observations of directional neurite outgrowth closely resemble the predicted MNP motion calculated by mathematical modelling. This study highlights the benefits that magnetic control systems can have on directing neuronal cell behaviour and shows that this strategy can form a useful component of cell therapy toolkits.

## Data Availability

The data are provided at https://doi.org/10.5281/zenodo.7217515 [33] and in the electronic supplementary material [34].

## References

[1] Castelo-Branco G et al. 2003 Differential regulation of midbrain dopaminergic neuron development by Wnt-1, Wnt-3a, and Wnt-5a. Proc. Natl Acad. Sci. USA **100**, 12 747-12 752. (10.1073/pnas.1534900100) PMC240689 14557550

[2] Andersson ER et al. 2008 Wnt5a regulates ventral midbrain morphogenesis and the development of A9–A10 dopaminergic cells *in vivo*. PLoS ONE **3**, e3517. (10.1371/journal.pone.0003517) 18953410 PMC2568809

[3] Joksimovic M, Yun BA, Kittappa R, Anderegg AM, Chang WW, Taketo MM, McKay RDG, Awatramani RB. 2009 Wnt antagonism of *Shh* facilitates midbrain floor plate neurogenesis. Nat. Neurosci. **12**, 125-131. (10.1038/nn.2243) 19122665

[4] Tang M, Villaescusa JC, Luo SX, Guitarte C, Lei S, Miyamoto Y, Taketo MM, Arenas E, Huang EJ. 2010 Interactions of Wnt/β-catenin signaling and sonic hedgehog regulate the neurogenesis of ventral midbrain dopamine neurons. J. Neurosci. **30**, 9280-9291. (10.1523/JNEUROSCI.0860-10.2010) 20610763 PMC3578394

[5] Gordon T. 2010 The physiology of neural injury and regeneration: the role of neurotrophic factors. J. Commun. Disord. **43**, 265-273. (10.1016/j.jcomdis.2010.04.003) 20451212

[6] Raudzus F et al. 2020 Magnetic spatiotemporal control of SOS1 coupled nanoparticles for guided neurite growth in dopaminergic single cells. Sci. Rep. **10**, 1-15. (10.1038/s41598-020-80253-w) 33384447 PMC7775457

[7] Bray D. 1984 Axonal growth in response to experimentally applied mechanical tension. Dev. Biol. **102**, 379-389. (10.1016/0012-1606(84)90202-1) 6706005

[8] Fass JN, Odde DJ. 2003 Tensile force-dependent neurite elicitation via anti-β1 integrin antibody-coated magnetic beads. Biophys. J. **85**, 623-636. (10.1016/S0006-3495(03)74506-8) 12829516 PMC1303117

[9] Riggio C et al. 2014 The orientation of the neuronal growth process can be directed via magnetic nanoparticles under an applied magnetic field. Nanomed. Nanotechnol. Biol. Med. **10**, 1549-1558. (10.1016/j.nano.2013.12.008) 24407149

[10] Suter DM, Miller KE. 2011 The emerging role of forces in axonal elongation. Prog. Neurobiol. **94**, 91-101. (10.1016/j.pneurobio.2011.04.002) 21527310 PMC3115633

[11] Lowery LA, Van Vactor D. 2009 The trip of the tip: understanding the growth cone machinery. Nat. Rev. Mol. Cell Biol. **10**, 332-343. (10.1038/nrm2679) 19373241 PMC2714171

[12] Bongaerts M et al. 2020 Parallelized manipulation of adherent living cells by magnetic nanoparticles-mediated forces. Int. J. Mol. Sci. **21**, 1-20. (10.3390/ijms21186560) PMC7555211 32911745

[13] Schöneborn H, Raudzus F, Coppey M, Neumann S, Heumann R. 2018 Perspectives of RAS and RHEB GTPase signaling pathways in regenerating brain neurons. Int. J. Mol. Sci. **19**, 1-37. (10.3390/ijms19124052) PMC6321366 30558189

[14] Pankhurst QA, Connolly J, Jones SK, Dobson J. 2003 Applications of magnetic nanoparticles in biomedicine. J. Phys. D. Appl. Phys. **36**, R167-R181. (10.1088/0022-3727/36/13/201)

[15] Markides H, Rotherham M, El Haj AJ. 2012 Biocompatibility and toxicity of magnetic nanoparticles in regenerative medicine. J. Nanomater. **2012**, 13-15. (10.1155/2012/614094)

[16] Harrison R, Markides H, Morris RH, Richards P, El Haj AJ, Sottile V. 2017 Autonomous magnetic labelling of functional mesenchymal stem cells for improved traceability and spatial control in cell therapy applications. J. Tissue Eng. Regen. Med. **11**, 2333-2348. (10.1002/term.2133) 27151571 PMC5573958

[17] Markides H et al. 2018 Translation of remote control regenerative technologies for bone repair. npj Regen. Med. **3**, 1-12. (10.1038/s41536-018-0048-1) 29675269 PMC5904134

[18] Rotherham M, Nahar T, Goodman T, Telling N, Gates M, El Haj A. 2019 Magnetic mechanoactivation of Wnt signaling augments dopaminergic differentiation of neuronal cells. Adv. Biosyst. **3**, 1900091. (10.1002/adbi.201900091) 32648650

[19] Rotherham M, Haj AJE. 2015 Remote activation of the Wnt/β-catenin signalling pathway using functionalised magnetic particles. PLoS ONE **10**, 1-18. (10.1371/journal.pone.0121761) PMC4363733 25781466

[20] Malagelada C, Greene LA. 2008 PC12 cells as a model for Parkinson's disease research. In Parkinson's disease, pp. 375-387. Boston, MA: Elsevier.

[21] Greene LA, Tischler AS. 1982 PC12 Pheochromocytoma cultures in neurobiological research. *Advances in Cellular Neurobiology* **3**, 373-414. (10.1016/B978-0-12-008303-9.50016-5)

[22] Plan Sangnier A, Van de Walle AB, Curcio A, Le Borgne R, Motte L, Lalatonne Y, Wilhelm C. 2019 Impact of magnetic nanoparticle surface coating on their long-term intracellular biodegradation in stem cells. Nanoscale **11**, 16 488-16 498. (10.1039/C9NR05624F) 31453605

[23] Barnsley LC, Carugo D, Stride E. 2016 Optimized shapes of magnetic arrays for drug targeting applications. J. Phys. D. Appl. Phys. **49**, 225501. (10.1088/0022-3727/49/22/225501) 26458056

[24] Stoppini L, Buchs PA, Muller D. 1991 A simple method for organotypic cultures of nervous tissue. J. Neurosci. Methods **37**, 173-182. (10.1016/0165-0270(91)90128-M) 1715499

[25] Schöneborn H et al. 2019 Novel tools towards magnetic guidance of neurite growth: (I) guidance of magnetic nanoparticles into neurite extensions of induced human neurons and in vitro functionalization with RAS regulating proteins. J. Funct. Biomater. **10**, 32. (10.3390/jfb10030032) PMC6787644 31315182

[26] Semeano AT et al. 2022 Effects of magnetite nanoparticles and static magnetic field on neural differentiation of pluripotent stem cells. Stem Cell Rev. Rep. **18**, 1337-1354. (10.1007/s12015-022-10332-0) 35325357

[27] Gahl TJ, Kunze A. 2018 Force-mediating magnetic nanoparticles to engineer neuronal cell function. Front. Neurosci. **12**, 1-16. (10.3389/fnins.2018.00299) 29867315 PMC5962660

[28] Sierra-Fonseca JA, Najera O, Martinez-Jurado J, Walker EM, Varela-Ramirez A, Khan AM, Miranda M, Lamango NS, Roychowdhury S. 2014 Nerve growth factor induces neurite outgrowth of PC12 cells by promoting G*β*γ-microtubule interaction. BMC Neurosci. **15**, 132. (10.1186/s12868-014-0132-4) 25552352 PMC4302597

[29] Yun S et al. 2018 Design of magnetically labeled cells (Mag-Cells) for in vivo control of stem cell migration and differentiation. Nano Lett. **18**, 838-845. (10.1021/acs.nanolett.7b04089) 29393650

[30] Wheeler MA et al. 2016 Genetically targeted magnetic control of the nervous system. Nat. Neurosci. **19**, 756-761. (10.1038/nn.4265) 26950006 PMC4846560

[31] McRobbie DW. 2014 3.01 – Fundamentals of MR imaging. In Comprehensive biomedical physics (ed. A Brahme), pp. 1-19. Oxford, UK: Elsevier.

[32] Baker RR et al. 2022 Image-guided magnetic thermoseed navigation and tumor ablation using a magnetic resonance imaging system. Adv. Sci. **9**, 1-14. (10.1002/advs.202105333) PMC9036015 35106965

[33] Dhillon K et al. 2022 Directional control of neurite outgrowth: emerging technologies for Parkinson&s disease using magnetic nanoparticles and magnetic field gradients. Zenodo. (10.5281/zenodo.7217515) PMC9653228 36349444

[34] Dhillon K et al. 2022 Directional control of neurite outgrowth: emerging technologies for Parkinson's disease using magnetic nanoparticles and magnetic field gradients. Figshare. (10.6084/m9.figshare.c.6266161) PMC9653228 36349444

